# Subcritical cracking and sealing along cleavage planes and subgrain boundaries in plagioclase from the Mado Megamullion oceanic core complex

**DOI:** 10.1038/s41598-026-55578-7

**Published:** 2026-06-22

**Authors:** Kohei Nimura, Takamoto Okudaira, Katsuyoshi Michibayashi

**Affiliations:** 1 Department of Earth and Planetary Sciences, Graduate School of Environmental Studies, Nagoya University, Nagoya, 464-8601 Japan; 2 Department of Geosciences, Graduate School of Science, Osaka Metropolitan University, Osaka, 558-8585 Japan; 3 Research Institute for Marine Geodynamics, JAMSTEC, Yokosuka, 237-0061 Japan; 4 Present Address: National Institute of Polar Research, Tokyo, 190-8518 Japan

**Keywords:** Plagioclase, Stress corrosion, Subcritical cracking, Oceanic core complex, Lower oceanic crust, Crack–seal, Materials science, Solid Earth sciences

## Abstract

Subcritical cracking propagates fractures via stress-assisted chemical reactions at crack tips under stresses below the critical threshold. Although this mechanism is increasingly recognized as a driver of time-dependent damage accumulation and permeability evolution in the Earth’s crust, direct natural constraints on the lower oceanic crust remain limited. Here, we document plagioclase microstructural and compositional features consistent with subcritical cracking in a deformed oxide gabbro from the Mado Megamullion oceanic core complex, Shikoku Basin, Philippine Sea. We combine co-registered scanning electron microscopy, electron backscatter diffraction, and energy-dispersive X-ray spectroscopy mapping with MATLAB MTEX Toolbox processing to link plagioclase crystallographic orientation, misorientation, and anorthite content (An = Ca/(Ca + Na + K)). Anorthite content maps show heterogeneous low-An domains within plagioclase porphyroclasts. These low-An domains are locally subparallel to the trace of the (001) cleavage plane and locally contain subgrain boundaries. We interpret the low-An domains as possible crack–seal-related features formed by stress-corrosion-assisted subcritical cracking along cleavage planes and subgrain boundaries, followed by host-controlled epitaxial precipitation of sodic plagioclase. These grain-scale crack–seal features may indicate that permeability creation by cracking can be followed by permeability reduction through sealing in gabbros from oceanic core complexes at slow-spreading ridges.

**Supplementary Information:**

The online version contains supplementary material available at 10.1038/s41598-026-55578-7.

## Introduction

Brittle to semibrittle fracturing in the oceanic crust generates damage and transient permeability that enable hydrothermal circulation and chemical exchange, thereby modifying the transport properties and rock strength over time^1^. At slow-spreading mid-ocean ridges and back-arc basins, a significant component of plate separation may be accommodated by long-lived detachment faulting, which exhumes lower-crustal and upper-mantle materials to the seafloor, forming oceanic core complexes^2,3^. Microstructural studies of lower-crustal rocks from oceanic core complexes have documented that microfracturing can facilitate fluid–rock interaction and subsequent sealing in gabbroic rocks and may be linked to strain localization during detachment-related deformation^4,5^. In particular, plagioclase feldspar (NaAlSi_3_O_8_–CaAl_2_Si_2_O_8_), a major constituent of gabbroic rocks, can preserve deformation microstructures such as twins, subgrain boundaries, and healed microfractures in porphyroclasts^5^. Therefore, grain-scale constraints on cracking and crack sealing in plagioclase are essential for linking microstructural damage to permeability evolution and lower-crustal mechanical behavior^6–8^.

Subcritical cracking, also termed subcritical crack growth, is a time-dependent fracture mechanism in hydrous environments in which cracks propagate quasi-statically under stresses below the critical threshold^9–13^. In this regime, stress-assisted chemical reactions at crack tips, known as stress corrosion, can enhance crack propagation^11,14^. A substantial body of laboratory and theoretical work has established fracture mechanics descriptions of subcritical crack growth^12,15,16^, and time-dependent cracking is widely invoked as a key microphysical mechanism underlying brittle creep and other long-term deformation behaviors of rocks^17,18^. Recent experimental studies on feldspars have further demonstrated that chemically driven processes, including diffusion-mediated stress generation during alkali exchange, can sustain crack growth and influence cracking patterns^19,20^, highlighting the potential for a strong coupling between reaction progress, cracking, and transport pathways.

Despite these advances, direct natural constraints on subcritical cracking remain limited and debated^21–25^. Previous studies of plagioclase-bearing granitoid shear-zone rocks have shown that intragranular feldspar microcracks can be associated with crack-tip reactions, grain-size reduction, and fracture-filling growth of secondary feldspar, including locally more sodic plagioclase compositions^21,22,24^. These observations provide important evidence for stress-corrosion-related cracking and reaction-assisted sealing in feldspar-rich continental rocks. In lower oceanic crustal gabbros, similar coupling among microcracking, fluid infiltration, and reactions is expected because hydrothermal fluids commonly infiltrate plagioclase-bearing gabbroic rocks^3,5^. However, direct grain-scale evidence remains limited because samples from the lower oceanic crust are difficult to access^26^, and early crack-related features can be overprinted by subsequent deformation, fluid infiltration, and metamorphic reactions^5^. Therefore, it remains unclear how chemically assisted cracks localize within plagioclase in gabbroic lower-crustal rocks, particularly with respect to crystallographic or microstructural weak planes and subsequent sealing.

**Fig. 1:**
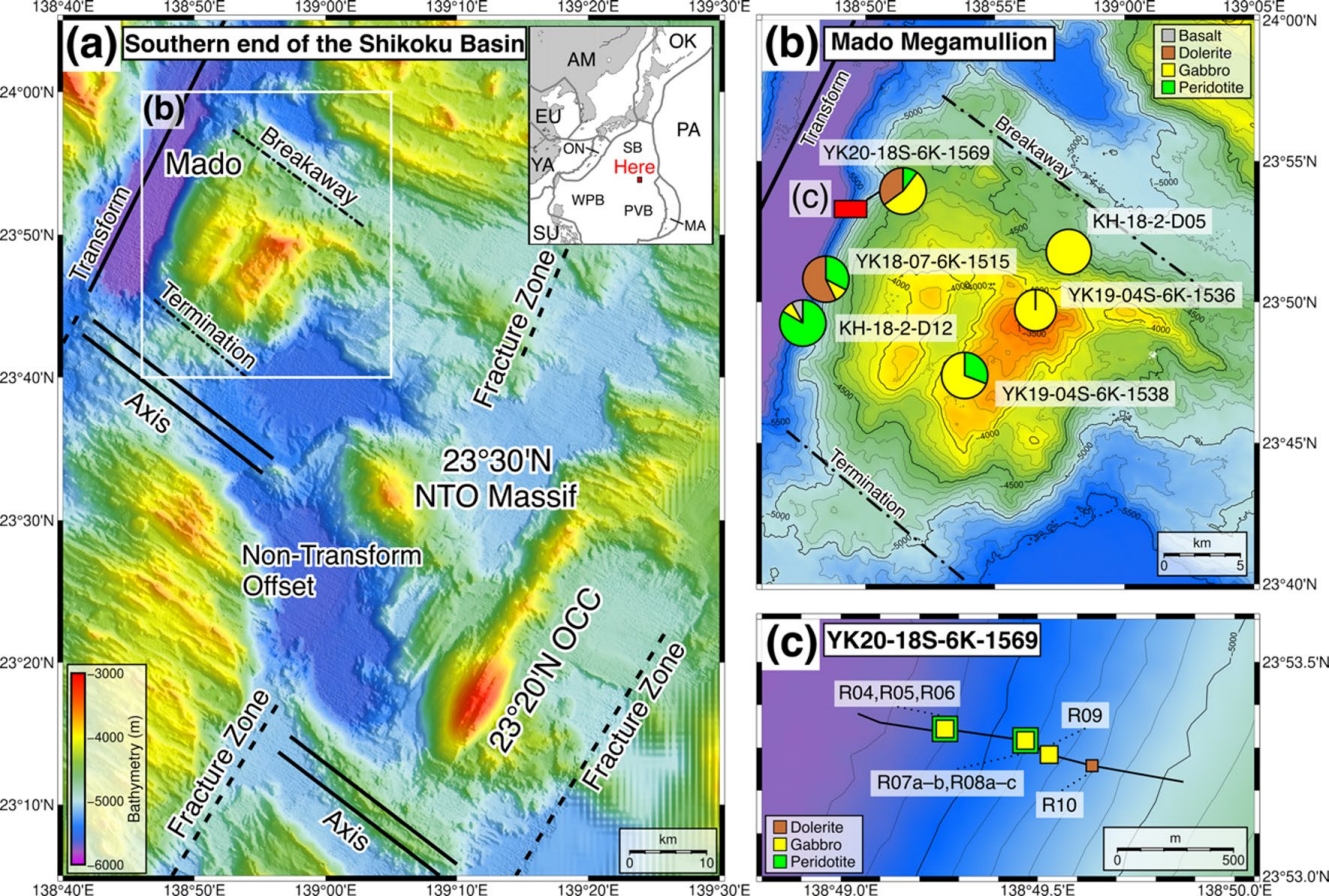
Bathymetric and lithological maps of the Mado Megamullion, Shikoku Basin, Philippine Sea. (**a**) Bathymetric map of the studied area. Top right inset shows the plate boundaries. AM: Amurian Plate, EU: Eurasia Plate, MA: Mariana Trough, OK: Okhotsk Plate, ON: Okinawa Trough, PA: Pacific Plate, PVB: Parece Vela Basin, SB: Shikoku Basin, SU: Sunda Plate, WB: West Philippine Basin, YA: Yangtze Plate. (**b**) Lithological map of the Mado Megamullion. Pie charts show the estimated relative abundance of the sampled lithologies (wt%). (**c**) Lithological map of the YK20-18S-6K-1569 site; the black line indicates the *Shinkai 6500* dive track. The maps were plotted using The Generic Mapping Tools (GMT) version 6.6.0^54^(https://www.generic-mapping-tools.org; accessed on May 24, 2026).

In this study, we developed a MATLAB MTEX Toolbox-based workflow to jointly process misorientation and anorthite content (An = Ca/(Ca + Na + K)) maps for plagioclase in a deformed oxide gabbro from the Mado Megamullion oceanic core complex, Shikoku Basin, Philippine Sea (Fig. 1). Using co-registered scanning electron microscopy (SEM), electron backscatter diffraction (EBSD), and energy-dispersive X-ray spectroscopy (EDS) mapping, we directly linked compositional domains to crystallographic orientation, twin boundaries, and subgrain boundaries. We evaluated fluid-assisted cracking along crystallographic weak planes, including cleavage planes and subgrain boundaries, and discussed its implications for brittle deformation and fluid–rock interactions in oceanic core complexes.

## Geological setting

Mado Megamullion is an oceanic core complex located at the inside corner of a ridge–transform intersection along the southernmost spreading axis of the Shikoku Basin (23°40′–60′ N, 138°45′–139°05′ E; Fig. 1)^3,27–33^. It is characterized by spreading-parallel corrugations extending for ~ 24 km from the distal breakaway region to the proximal termination region. The corrugated surface covers ~ 550 km^2^ and exposes serpentinized peridotites, variably evolved and deformed gabbroic rocks, and minor basaltic and doleritic rocks (Fig. 1). It is also characterized by a positive mantle Bouguer anomaly^32^. These bathymetric, lithological, and geophysical features are consistent with those of typical oceanic core complexes at mid-ocean ridges^34^.

Mado Megamullion is estimated to have formed from 14.1 to 12.2 Ma during the final stage of Shikoku Basin opening^32^. Based on petrological and geochemical analyses, the following magmatic and tectonic history of the Mado Megamullion has been proposed^27,29,30^: (1) deep-seated formation of the dunites and gabbros, and formation of the detachment fault; (2) intrusion of mafic to felsic plutonic rocks during progressive exhumation of the Mado Megamullion; and (3) intrusion of dolerite dykes after the final emplacement of the Mado Megamullion (9.7 ± 6.3 Ma)^30^.

The geochemical and microstructural characteristics of the recovered gabbroic rocks from the Mado Megamullion resemble those of oceanic core complexes at slow- to ultraslow-spreading ridges^3,27,29^. These gabbroic rocks are interpreted to have crystallized from highly evolved, hydrous ferrobasaltic melts, reflecting progressive water enrichment during magma differentiation beneath the extinct spreading center of the Shikoku Basin^27,29^. Previous microstructural work further suggests that fluid-assisted ductile deformation, including dissolution–precipitation processes and grain-boundary sliding, contributed to strain localization in lower-crustal gabbros, similar to deformation processes documented in oceanic core complexes at mid-ocean ridges^3^.

**Fig. 2:**
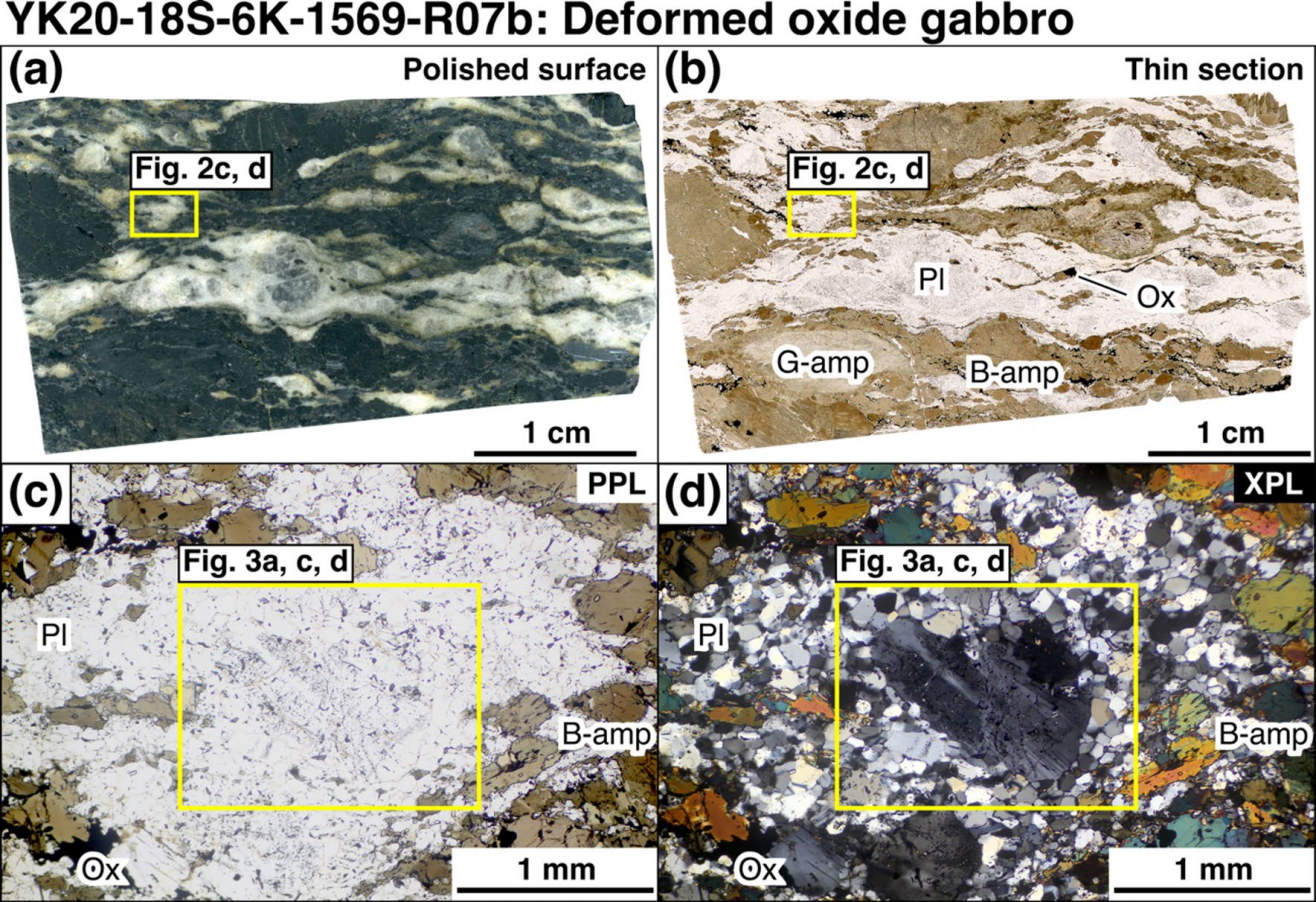
Sample YK20-18S-6K-1569-R07b. (**a**) Photograph of the polished sample. (**b**) Photograph of the thin section. Yellow squares in (a) and (b) indicate the area shown in (c) and (d). (c, d) Photomicrographs showing the plagioclase porphyroclast and surrounding matrix analyzed in this study. (**c**) Plane-polarized light (PPL). (**d**) Cross-polarized light (XPL). Yellow squares in (c) and (d) indicate the area shown in Fig. 3a, c, and d. B-amp, brown amphibole; G-amp, green amphibole; Ox, Fe–Ti oxides; Pl, plagioclase.

The sample analyzed in this study is a deformed oxide gabbro from dive site YK20-18S-6K-1569 (23°53.3168′ N, 138°49.4689′ E, 5422 m water depth), located on a west-dipping slope in the medial region of the Mado Megamullion, near the transform fault (Fig. 1). During cruise YK20-18S aboard the R/V *Yokosuka*, the submersible *Shinkai 6500* collected 10 samples from this locality^29,33^, including 1 dolerite, 6 gabbroic rocks, and 3 ultramafic rocks together with some pumice (Fig. 1c). From this suite, we selected the deformed oxide gabbro YK20-18S-6K-1569-R07b for detailed microstructural analyses because it contains well-preserved plagioclase porphyroclasts suitable for investigating subcritical cracking and crack–sealing processes (Figs. 1 and 2).

## Results

### Microstructures and mineral compositions of the analyzed oxide gabbro

Sample YK20-18S-6K-1569-R07b consists of plagioclase (52.1 vol%), brown amphibole (33.5 vol%), green amphibole (10.4 vol%), Fe–Ti oxides (3.5 vol%), and minor clinopyroxene (0.5 vol%) (Fig. 2a, b). The sample shows a porphyroclastic texture (Fig. 2a, b). A foliation is defined by monomineralic layers of plagioclase and amphibole (Fig. 2b). Plagioclase porphyroclasts are ~ 1 mm across and show deformation twinning and subgrain boundaries (Fig. 2c, d). The matrix is dominated by fine-grained plagioclase with polygonal grain boundaries (Fig. 2c, d).

Plagioclase porphyroclast cores show higher anorthite contents (An_42_–An_46_; andesine) than fine-grained matrix plagioclase (An_16_–An_40_; oligoclase–andesine) (see Methods and Supplementary Table S1 online). Brown amphibole cores show magnesio-hornblende compositions, whereas green amphibole cores show magnesio-hornblende to tremolite compositions (see Methods and Supplementary Table S2 online). Brown amphibole rims adjacent to fine-grained matrix plagioclase show magnesio-hornblende compositions, whereas green amphibole rims adjacent to fine-grained matrix plagioclase show pargasite to magnesio-hornblende compositions (see Methods and Supplementary Table S2 online). Equilibrium temperatures calculated from these amphibole rims and adjacent fine-grained matrix plagioclase rims (An_20_–An_29_; oligoclase) range from 684 to 755 °C (see Methods and Supplementary Table S3 online).

### SEM–EBSD–EDS observations

**Fig. 3:**
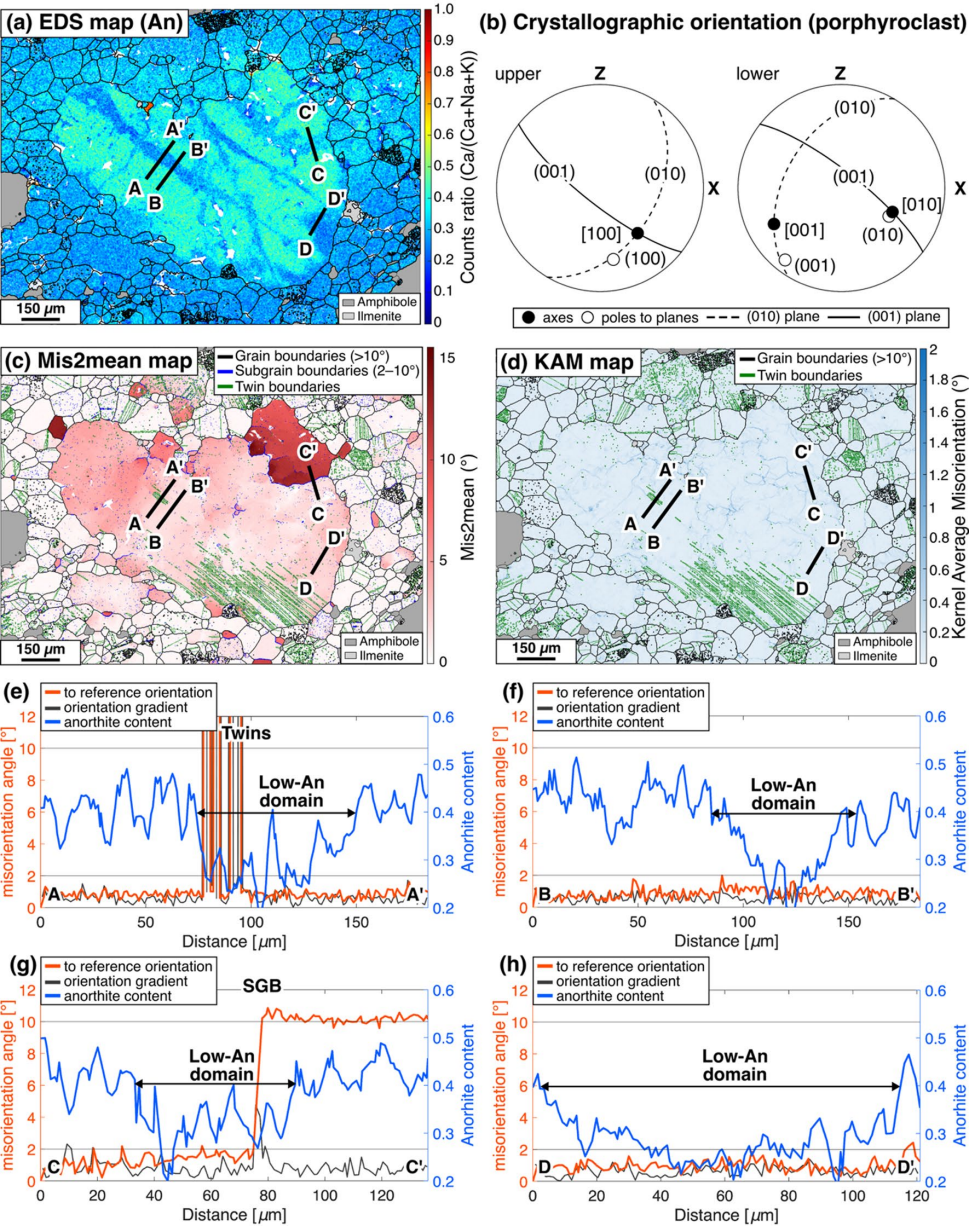
Results of SEM–EBSD–EDS analyses of plagioclase in sample YK20-18S-6K-1569-R07b (see Methods). Grain boundaries shown in (a), (c), and (d) were drawn from the twin-merged grain dataset. (**a**) EDS-derived anorthite content (An) map (An = Ca/(Ca + Na + K)). (**b**) Crystallographic orientation of the plagioclase porphyroclast. Upper- and lower-hemisphere equal-area stereonets are shown. Open symbols indicate poles to planes; solid symbols indicate axes. Representative cleavage planes, the (010) and (001) planes, are indicated by dashed and solid lines, respectively. (**c**) Mis2Mean map calculated from the unmerged EBSD dataset. Black lines, grain boundaries; blue lines, subgrain boundaries (SGBs); green lines, twin boundaries. The observed twin boundaries mostly correspond to pericline twins. (**d**) Kernel average misorientation (KAM) map calculated from the denoised, unmerged EBSD dataset using first-order neighbors and a 2° threshold. (**e–h**) Profiles of misorientation angle and An content along transects across low-An domains. Orange, dark gray, and blue lines indicate misorientation to reference orientation (A, B, C, and D), orientation gradient, and An content, respectively. Transect A–A′ crosses twin boundaries; B–B′ crosses neither twin boundaries nor SGBs; C–C′ crosses an SGB; and D–D′ crosses a low-An domain near the porphyroclast rim without twin boundaries or SGBs.

We focused our SEM–EBSD–EDS observations on the plagioclase porphyroclast and surrounding fine-grained matrix shown in Fig. 2c, d. The resulting maps show spatial variations in plagioclase anorthite content and crystallographic orientation (Fig. 3; see Methods). Anorthite content (An = Ca/(Ca + Na + K)) was calculated from EDS Ca, Na, and K count maps (Fig. 3a), whereas crystallographic orientation and related boundary/misorientation features were derived from EBSD orientation data (Fig. 3b–d). The porphyroclast has a higher An content than the surrounding fine-grained matrix (Fig. 3a). Local low-An domains occur as vein-like features within the porphyroclast (Fig. 3a). Comparison with the crystallographic orientation of the plagioclase porphyroclast shown in the stereonets (Fig. 3b) and the EBSD-derived misorientation map (Fig. 3c) shows that some of these vein-like features are oriented subparallel to the trace of the (001) cleavage plane and to twin boundaries (Fig. 3b, c).

Misorientation and boundary distributions differ between the porphyroclast and the fine-grained matrix (Fig. 3c). Mis2Mean values (misorientation to mean orientation) are higher in the porphyroclast than in the fine-grained matrix (Fig. 3c). Subgrain boundaries (2–10°) are more commonly developed within the porphyroclast than within the fine-grained matrix (Fig. 3c). Most identified twin boundaries correspond to pericline twins (Fig. 3c). In some areas of the porphyroclast, low-An domains contain subgrain boundaries (Fig. 3c). The kernel average misorientation (KAM) map further shows local misorientation gradients below 2° within the porphyroclast (Fig. 3d). These local misorientation gradients are locally associated with low-An domains and occur near some subgrain boundaries (Fig. 3a, c, d).

Line profiles further show the variable relationships between low-An domains, twin boundaries, and subgrain boundaries within the porphyroclast (Fig. 3e–h). Transect A–A′ crosses a ~ 80 μm-wide low-An domain containing twin boundaries at a distance of ~ 80 μm along the profile (Fig. 3e). Transect B–B′ crosses the same low-An domain, which is ~ 60 μm wide along the transect and contains neither twin boundaries nor subgrain boundaries (Fig. 3f). In both cases, the low-An domain shows an anorthite content of 0.2–0.4, regardless of the presence of twin boundaries. Transect C–C′ crosses a low-An domain containing a subgrain boundary, marked by an abrupt change in the misorientation angle at ~ 80 μm (Fig. 3g). Transect D–D′ crosses a low-An domain at the rim of the porphyroclast, which contains neither twin boundaries nor subgrain boundaries (Fig. 3h). Along D–D′, the change in anorthite content is gradual (Fig. 3h).

## Discussion

Low-An plagioclase occurs both in the fine-grained matrix surrounding the porphyroclast and as localized domains within the porphyroclast (Fig. 3a; Supplementary Table S1). These two occurrences likely reflect different formation processes. The surrounding fine-grained matrix is characterized by lower An contents (An_16_–An_40_; oligoclase–andesine) than the porphyroclast cores (An_42_–An_46_; andesine) (Supplementary Table S1), lower Mis2Mean values, and fewer subgrain boundaries (Fig. 3c). Therefore, these results suggest that the fine-grained plagioclase matrix was produced mainly by metamorphic recrystallization in the presence of fluids^3,35^. Amphibole rims adjacent to fine-grained matrix plagioclase show pargasite to magnesio-hornblende compositions and are more Al-rich than green amphibole cores (Supplementary Table S2). This compositional relationship is consistent with limited Al mobility during amphibole-forming hydration reactions, in which relatively Al-rich amphibole can form or be preserved near plagioclase as an Al source^36^. Equilibrium temperatures of 684–755 °C calculated from amphibole rims and adjacent fine-grained matrix plagioclase rims may therefore represent the temperature of hydration-related recrystallization of the fine-grained matrix (Supplementary Table S3).

Low-An domains within the porphyroclast occur as narrow, planar to vein-like features with locally irregular boundaries (Fig. 3a). These features differ from the surrounding fine-grained matrix and from simple growth zoning. Similar intragranular feldspar microstructures in granitoid shear-zone rocks have been interpreted as sealed microcracks or crack-related reaction domains^21,22,24^. By analogy, we interpret the low-An vein-like domains in the present sample as possible sealed crack-related pathways for fluid infiltration and subsequent chemical modification.

**Fig. 4:**
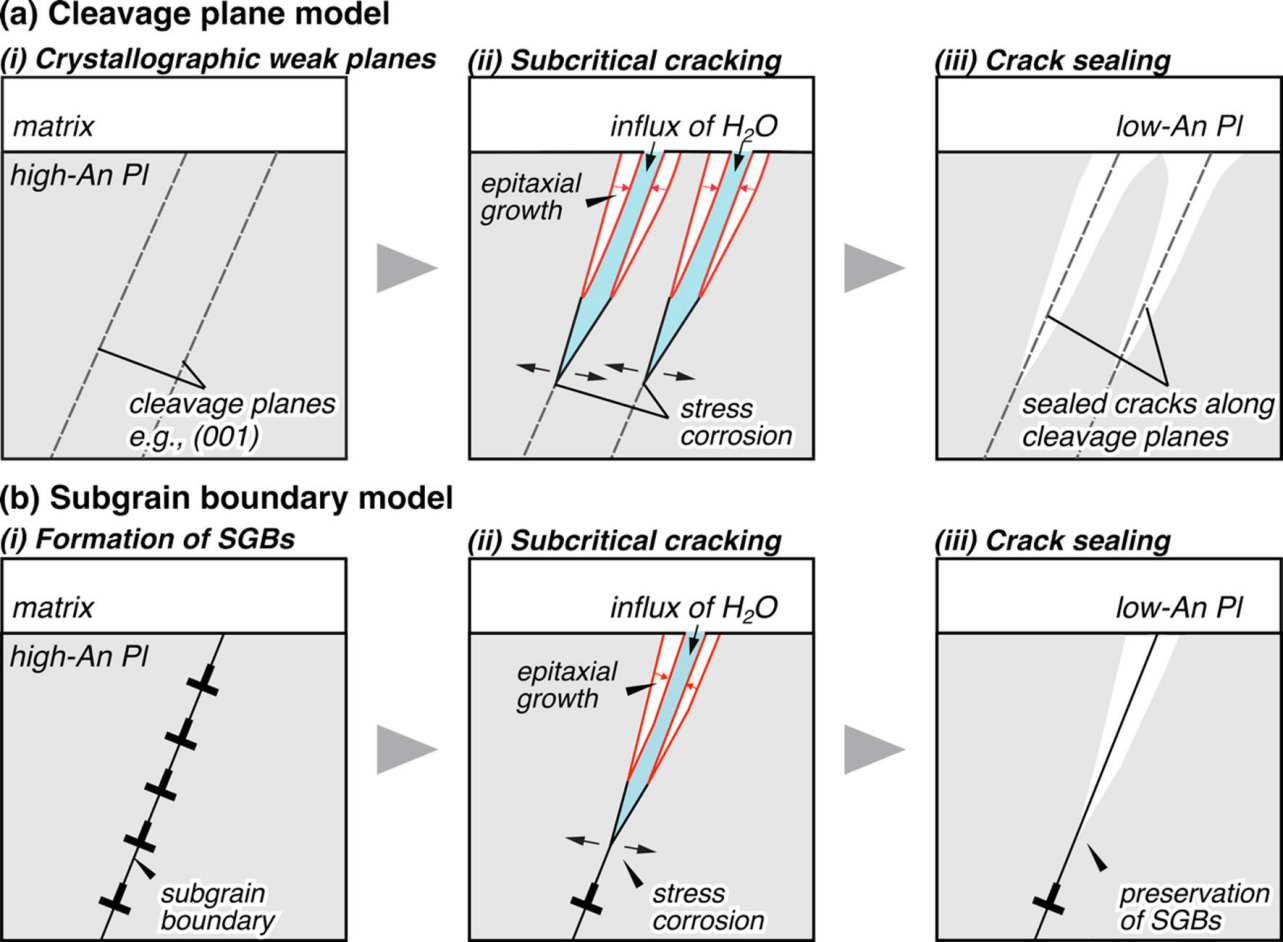
Model illustrating subcritical cracking and sealing along cleavage planes and subgrain boundaries (SGBs), modified after Yoshioka et al.^25^. In both models, water preferentially diffuses along crystallographic or microstructural weak planes, promoting stress-corrosion crack propagation, dissolution of high-An plagioclase, and subsequent epitaxial precipitation of low-An plagioclase. (**a**) Cleavage plane model: (i) presence of crystallographic weak planes, e.g., (001) plane; (ii) crack propagation along cleavage planes; and (iii) crack sealing along cleavage planes. Twin boundaries may form after sealing. (**b**) Subgrain boundary model: (i) formation of an SGB with dislocation accumulation (denoted by ‘⊥’); (ii) crack propagation along the SGB; and (iii) crack sealing, after which the SGB may remain preserved.

Some low-An domains within the porphyroclast are oriented subparallel to the trace of the (001) cleavage plane of plagioclase (Fig. 3a, b), one of the representative cleavage planes in plagioclase^37^. Transect A–A′ crosses a low-An domain containing twin boundaries, whereas transect B–B′ crosses the same low-An domain and contains neither twin boundaries nor subgrain boundaries (Fig. 3e, f). In both cases, the low-An domains show similar An contents of 0.2–0.4, regardless of the presence of twin boundaries. These observations indicate that the low-An domains are not necessarily controlled by pre-existing twin boundaries. Instead, they are more plausibly interpreted as crack-related features formed along crystallographic weak planes, including cleavage planes such as the (001) plane (Fig. 4a). Experimental deformation of plagioclase has shown that microfracturing and mechanical twinning can interact during deformation in the brittle–plastic transition^38^. Thus, the presence of twin boundaries in some low-An domains may reflect either local preservation of twin-related structures or later formation of twins during continued deformation after crack sealing.

In contrast, the domain crossed by transect C–C′ contains a subgrain boundary (Fig. 3g), indicating that subgrain boundaries can remain preserved within low-An domains after sealing (Fig. 4b). The KAM map also shows local misorientation gradients below 2° within and around some low-An domains, locally near subgrain boundaries (Fig. 3a, c, d), suggesting that these domains can be associated with local intracrystalline distortion as well as discrete subgrain boundaries. Subgrain boundaries, which are commonly represented by dislocation walls, may also have acted as microstructural weak planes for crack initiation and propagation^25^. When plagioclase precipitates from fluids within cracks, the crystallographic orientation of the newly formed material can be influenced by the adjacent host plagioclase through epitaxial growth^39^. If growth proceeds from one or both crack walls, sharp orientation changes can be preserved within the crack-filling material or at the interfaces where the growth domains impinge. Therefore, we interpret the low-An vein-like domains as crack-related features formed along cleavage planes and/or subgrain boundaries, followed by the precipitation of sodic plagioclase from crack-filling fluids (Fig. 4).

The low-An domains further imply Na enrichment in plagioclase, consistent with albitization during fluid–rock interaction^3^. Although the exact reaction pathway, fluid composition, and silica activity are not directly constrained in the present sample, the decrease in An content indicates fluid-assisted dissolution or chemical modification involving enrichment in the albite component relative to the surrounding higher-An plagioclase (Fig. 4). Coupled with the spatial relationships between low-An domains, twin boundaries, and subgrain boundaries, this Na enrichment supports a scenario in which fluid-assisted reactions promoted crack propagation, followed by precipitation of sodic plagioclase during crack filling^21,22,24^. These features are consistent with stress-corrosion-assisted cracking (Fig. 4), in which fluid–mineral reactions facilitate subcritical crack growth and can be coupled with syn- to post-cracking mineral precipitation^25^.

Alternative explanations for the low-An bands include magmatic or metamorphic zoning in plagioclase^40,41^, CaAl–NaSi lattice interdiffusion^42^, and fluid-mediated interface-coupled replacement reactions during fluid–rock interactions^39^. However, magmatic or metamorphic zoning would generally be expected to produce systematic compositional variations within a grain^40,41^. Such systematic zoning patterns were not observed in the studied porphyroclasts; instead, low-An domains are heterogeneously distributed as localized vein-like bands within porphyroclasts (Fig. 3a). If CaAl–NaSi interdiffusion were the dominant process, porphyroclasts would be expected to show relatively smooth compositional gradients near the margins driven by exchange with the surrounding, more sodic matrix. Such gradients were not observed, except for the low-An domain at the porphyroclast rim, which lacks both twin and subgrain boundaries and shows a more gradual decrease in An content (Fig. 3h). This suggests that some marginal modifications may have involved diffusional exchange. Interface-coupled replacement can account for albitization in feldspar and has been described to progress via a migrating reaction front in unfractured grain domains^39^. In contrast, the strongly patchy, vein-like geometry of the low-An bands in our porphyroclasts, together with their variable spatial relationships with cleavage planes and subgrain boundaries (Fig. 3), is difficult to reconcile with a simple grain-margin-inward replacement front and instead supports cracking-controlled fluid infiltration and localized precipitation.

Our microstructural and compositional data are consistent with stress-corrosion-assisted subcritical cracking and subsequent crack sealing in plagioclase from the lower oceanic crust. The occurrence of low-An, vein-like domains subparallel to the trace of the (001) cleavage plane and containing subgrain boundaries in some cases, together with the host-controlled epitaxial growth of sodic plagioclase, suggests that cleavage planes and subgrain boundaries can localize fluid access, crack propagation, and crack filling at the grain scale. This crack–seal behavior controlled by crystallographic weak planes and subgrain boundaries offers a microphysical explanation for the accumulation of brittle damage under subcritical conditions while preserving only subtle compositional and microstructural fingerprints in plagioclase.

Our findings suggest that these grain-scale crack–seal features may represent a mechanism for time-dependent permeability evolution in detachment-related lower-crustal gabbros, in which permeability creation by fracturing competes with permeability loss by sealing. In an in-situ fault zone from the Atlantis Massif (Mid-Atlantic Ridge), permeability created during high-temperature brittle failure at about 640 °C was inferred to be rapidly reduced by extensive sealing by hydrous minerals, limiting subsequent fluid circulation^4^. At the Atlantis Bank (Southwest Indian Ridge), microfracturing facilitated pervasive fluid–rock interactions and contributed to reaction weakening in ultramylonites^5^. Permeability may also increase through reaction-induced fracturing during hydration reactions at low temperatures (< 350 °C), which can generate connected fracture networks and enhance fluid penetration through initially low-permeability lower crust, as reported from the Oman ophiolite^43^. Conceptual models further suggest that the balance between crack sealing and crack opening depends on permeability, strain rate, and fluid overpressure^25^. In this framework, the presence of crack-filling sodic plagioclase in our sample (Fig. 3) is consistent with sealing under comparatively low strain rates and/or elevated fluid overpressure^25^, and thus with a permeability trajectory toward reduction as sealing progresses, in line with the Atlantis Massif-style context of rapid post-fracture sealing^4^. Therefore, our results place stress-corrosion-assisted subcritical cracking among the plausible grain-scale microphysical processes that can generate and seal intragranular cracks in plagioclase, with implications for time-dependent permeability evolution in lower-crustal gabbros from oceanic core complexes at slow-spreading ridges.

## Methods

### Sample preparation

Microstructures were examined in thin sections cut as close as possible to perpendicular to the foliation and parallel to the lineation (i.e., XZ sections).

### Major element composition analysis of plagioclase and amphibole

The major element compositions of plagioclase and amphibole grains were obtained using an electron probe microanalyzer (EPMA) equipped with wavelength- and energy-dispersive X-ray systems (JXA-8900R) at the Rock and Mineral Laboratory, Nagoya University (see Supplementary Tables S1 and S2 online). The operating conditions were a probe current of 12 nA, an accelerating voltage of 15 kV, and a beam diameter of 1 μm. Corrections were performed following Bence and Albee^44^. Amphibole nomenclature follows the classification scheme of Leake et al.^45^.

### Amphibole–plagioclase geothermometry

Rim compositions of amphibole and adjacent fine-grained matrix plagioclase were analyzed to estimate equilibrium temperatures (see Supplementary Table S3 online). Temperatures were calculated using the amphibole–plagioclase geothermometer of Holland and Blundy^46^. A pressure of 200 MPa, corresponding to the mid-crustal level, was assumed. We used this thermometer because it is calibrated for both silica-saturated and silica-undersaturated rocks over a temperature range of 500–900 °C and for plagioclase compositions of An_10_–An_90_. The uncertainty of this thermometer is 35–40 °C^46^.

### Analytical considerations for SEM–EBSD–EDS mapping

The combination of EBSD and EDS acquisition provides a pixel-scale correlation; however, it introduces practical and analytical constraints. Nowell and Wright^47^ demonstrated that chemical information (XEDS) acquired simultaneously can be used with EBSD to improve phase differentiation. They also discussed limitations relevant to mapping, such as a spatial-resolution mismatch between XEDS (order of 1–2 μm) and EBSD (down to tens of nanometers) and long-range count gradients caused by the ~ 70° tilt geometry. They noted that using the ratios of region-of-interest counts can reduce the sensitivity to absolute intensity gradients. In materials science, several studies have proposed systematic approaches to integrate EBSD and EDS for phase discrimination and segmentation^48,49^. In contrast, comparable standardized workflows for natural minerals are still under development, although recent studies have begun to correlate compositional and microstructural information at the grain scale in deformed mafic rocks^50^. Based on these considerations, we designed our analytical and data-processing workflow to evaluate relative compositional variations in plagioclase and their spatial relationships with crystallographic and microstructural features.

### SEM–EBSD–EDS analytical procedures

Integrated SEM–EBSD–EDS analyses were used to map the plagioclase misorientation and composition (Fig. 3). Analyses were performed at Nagoya University using a Hitachi S-3400N SEM equipped with an Oxford Instruments EBSD detector. A pre-tilted holder was used, resulting in a specimen tilt of 70° during the EBSD measurements. For the EDS acquisition, the detector was partially inserted into a position near the maximum count rate. The accelerating voltage was 20 kV under low-vacuum conditions, with a chamber pressure of 30 Pa. The step size was 1.59 μm.

### SEM–EBSD–EDS data processing

The combined EBSD–EDS datasets were cleaned using the EBSDinterp toolbox for MATLAB^51^. Non-indexed pixels were filled when three or more neighbors shared the same orientation. Band-contrast masking was applied to restrict the interpolation to high-quality patterns^52^. Wild-spike pixels were removed during this cleaning procedure. The maps were processed and plotted using the open-source MTEX toolbox for MATLAB (version 5.11.2)^53^. The grains were identified using a critical misorientation of 10°, and those containing fewer than four pixels were discarded. Twin-related domains were subsequently merged to define host grains and grain boundaries for map overlays. Grain boundaries shown in Fig. 3a, c, and d were drawn from this twin-merged grain dataset. Mis2Mean values were calculated from the unmerged EBSD dataset before merging twin-related domains, so that twin-related orientation domains and subgrain-scale misorientation within the porphyroclast could be visualized. Before calculating kernel average misorientation (KAM), the EBSD orientation map was denoised using a half-quadratic filter in MTEX with alpha = 0.5. KAM values were then calculated from the denoised, unmerged EBSD dataset using first-order neighbors and a threshold angle of 2°, so that local misorientation gradients below the subgrain-boundary threshold could be visualized. Subgrain boundaries were defined using a misorientation threshold of 2°. Twin boundaries were identified separately based on boundary misorientation angles of > 170° and rotation axes corresponding to the major plagioclase twin laws: [010] for pericline twins, [001] for Carlsbad twins, and the direction normal to the (010) plane for albite twins.

For EDS processing, elemental count maps were denoised using the MATLAB command smoothdata2 with a Gaussian-weighted moving average (method = “gaussian”). The anorthite content (An) maps were calculated as An = Ca/(Ca + Na + K) from smoothed Ca, Na, and K count maps.

## Supplementary Information

Below is the link to the electronic supplementary material.

Supplementary Material 1

## Data Availability

The datasets generated and/or analyzed during the current study are available from the corresponding author upon reasonable request.
